# Therapeutical and Pharmaceutical Notes

**Published:** 1901-04

**Authors:** W. J. Martin

**Affiliations:** Kankakee, Ill.


### THERAPEUTICAL AND PHARMACEUTICAL NOTES.

UNDER THE DIRECTION OF W. J. MARTIN, M.D.C.,
KANKAKEE, ILL.

---

A DISASTROUS EXPLOSION. The results of the explosion of large quantities of sulphur and chlorate of potassium stored in the wholesale drug establishment of Tarrant & Co., of New York City, were simply appalling. Seven lives were lost, ten buildings with their contents were totally destroyed, thirty-five other buildings were considerably damaged, and two hundred others had windows and plate glass broken by the concussion. The loss by fire and explosion is estimated at $1,000,000.

It is supposed that the explosion of the chlorate of potassium was due to the sudden liberation of oxygen gas by heat and smoke. Tarrant & Co. have been found criminally responsible by the Coroner's jury for the disaster, by storing chemicals in the building in excess of that permitted by law. It was shown by the testimony that over 32,000 pounds of sulphur and about thirty-five tons of chlorate of potassium were stored in the building at the time the fire took place.

LOOSEN THE CORK. An account of another hydrogen peroxide explosion was recently given in a Canadian journal. A bottle of the solution standing on a shelf burst by the decomposition of the contents, and surrounding objects were seriously damaged. Fortunately no one was struck by the flying débris. The *Druggists' Circular* thinks this accident should serve as a warning against the risk of storing hydrogen peroxide solution in the ordinary way—a risk that is not avoided by keeping the solution cool and in a dark place, as has been suggested. "The only assurance of safety," says the *Circular*, "is in leaving the cork of the bottle so loose that it will be easily displaced by a slight pressure of liberated gas. It is well known that solutions of hydrogen dioxide are prone to decomposition, and there is no evidence to show that keeping tightly corked has any preservative effect. And when decomposition ensues there is no use whatever in retaining the gas, which is simply oxygen, and cannot be again forced into combination by pressure. Whatever one may think as to the relative permanence of this or that make of the solution, it will do no harm to leave the stopper loose, and this loosening may prevent a serious or fatal accident.—*Bull. Pharm.*

ALCOHOL AS A DISINFECTANT. Drs. Salzwedel and Elsner assert that alcohol has a marked disinfecting action, which is best exerted in strengths of 50 to 55 per cent. Stronger spirit has more coagulative power, and hence may fail to get at the microbes. Much weaker solutions delay the growth of diseased germs in the same way as that of yeast. The authors hold that their experiments show that alcohol is of use in preparing the hands of the doctor for operations, not merely because of its hardening effects on the skin, but also as an active antiseptic. They assign to this disinfectant, says the *British Medical Journal*, a potency intermediate between that of carbolic acid and corrosive sublimate, though it is, of course, much less poisonous than either and usually more handy.

SCARCITY OF SPONGES. The scarcity of sponges gradually grows as time passes. Just what will be the outcome no one ventures to say. At the present rate sponges will soon be a luxury only within the reach of the wealthier classes. At the present time there is an unusual scarcity due to special conditions, among which may be mentioned hurricanes in the West Indies, which have rendered the waters muddy and prevent the usual method of fishing with long poles. The trouble between Turkey and Greece also interfered with the output of the higher grades of goods. It remains for some ingenious American to discover a cheap and acceptable substitute for the skeleton of the sponge animal.—*Myers Brothers' Druggist.*

RESISTANCE OF BACTERIA TO HEAT AND COLD. Dr. A. McFadyean finds (*Lancet*) that bacteria may be kept at a temperature of 190° C. for twenty-four hours without their vital powers being affected. The organisms with which he experimented possessed varying degrees of resistance to external agents, the extremes being represented by the very sensitive sporillum of cholera Asiatica, and the highly resistant spores of the anthrax bacillus. Pure cultures were taken of bacillus typhosus, B. coli communis, B. diphtheria, S. cholera Asiatica, B. proteus vulgaris, B. acidi lactici, B. anthracis (sporing culture), staphylococcus pyogenes aureus, B. phosphorescens, and photobacterium balticum. They were simultaneously exposed to the temperature of liquid air (182° C. to 190° C.) for twenty hours, then carefully thawed and examined. In no instance could any impairment of vitality be detected, the fresh growth obtained being normal in every respect, and the functional activities of the bacteria quite unaffected.

Experiments with representative types of organisms usually met with in the air—moulds, bacilli, cocci, torulæ, and sarcinæ—had similar results, while a sample of yeast-cell plasma (Buchner's zymase) retained its peculiar properties unchanged as regards the production of carbon dioxide and alcohol after twenty-four hours' exposure to the intense cold mentioned.

TUBERCULIN "R." The *Medical Standard* thus defines the difference between tuberculin "R." and other similar preparations: "The old tuberculin is made by growing tubercle bacilli in bouillon, concentrating the fluid by heat to one-tenth of its original volume and filtering the serum; the clear filtrate is used. The new tuberculin is made from the bodies of the bacilli themselves. These are first reduced to powder and the solution centrifugalized, when the liquid is separated into two layers. Koch called the upper layer tuberculin O. (from obere—upper). The lower layer was called tuberculin R. (residue). This was insoluble in glycerin, and when injected gave a different reaction from either the old tuberculin or tuberculin O., which were very much alike."

PRESERVATIVES IN ANTITOXIN. An article by Dr. George F. Cott in the *American Therapist* gives the following: Behring's contains ½ per cent. carbolic acid; Mulford's contains ½ per cent. of trikresol; Schering's contains 4 to 10 per cent. trikresol; Parke, Davis & Co.'s contains ½ per cent. trikresol; Buffalo contains ½ per cent. soda salicylate; Roux's (Paris) contains none. There are still some physicians who deny any curative effects to antitoxin, and ascribe all its good effects to the carbolic which it contains. To them it may be said that Roux's antitoxin, which contains no preservative, has a good reputation, while the toxic effects of carbolic acid have caused it to be generally replaced by trikresol, which is much less poisonous. Moreover, the tendency to use more concentrated serums with a consequent decrease in the amount of preservative shows that a large amount of the latter is not considered desirable. That the dangers of antitoxin are greatly overestimated is shown by the fact that only five deaths have followed its use in over 3,000,000 injections.—*Ibid.*

THE SIZE OF DROPS. According to M. R. A. Field (*Chemical News*), other conditions being the same, the size of drops varies with the rapidity of the dropping. A pipette, which delivered 136 drops at the rate of one in two seconds, delivered 141 at the rate of two a second. With increase of temperature, also, there is a notable decrease in the size of drops, even in the case of mobile fluids like water.

Pneumococcic Arthritis. Cave (*Lancet*), after studying thirty-one cases of pneumococcic arthritis, one of which he treated himself, observes that the association of the arthritis with other localizations of the pneumococcic infection is the rule; that this associated lesion is commonly in a vital part, and more directly threatening to life than is the arthritis. Besides the occurrence of pneumonia, in twenty-eight out of the thirty-one cases, there was a wide-spread infection of the system by the virus: with malignant endocarditis, six cases; pleurisy and empyema, five; pericarditis, two; nephritis, three; meningitis, six; and peritonitis, one. In some of the cases there was a simultaneous inflammation of more than one serous membrane. The morbid anatomy of the joint infected by pneumococci is essentially that of any other septic arthritis. In mild cases, or those of short duration, the synovial membrane alone may be affected, with less polish and injection of the fringes. In many, however, the cartilage is partly or completely eroded, and the surface of the bone is laid bare. In the older or more virulent cases the ligaments and cartilages, and in some the articular ends of the bones, are completely destroyed. Pus may perforate the capsule and penetrate several inches along the intermuscular planes or sheaths of tendons.

Ten cases of thirty-one studied gave direct evidence of the situation of the infection in a previously damaged joint—a fact of interest when it is compared with the experimental investigation of the pathogeny of the affection in animals.

The observations of numerous investigations are thus summarized: 1. The injection of a virulent culture of the pneumococcus directly into a joint of a susceptible animal is almost invariably followed by acute suppurative arthritis. 2. The subcutaneous injection of virulent cultures after the previous excitation of an aseptic inflammation in a joint may give varying results, either negative or positive. 3. The intravenous injection of virulent cultures with associated aseptic traumatism of a joint leads to arthritis much more certainly than do injections by the subcutaneous method. Bezancon and Griffon have succeeded in producing an experimental pneumococcic arthritis by modifying the resistance of the animal experimented on in relation to the virulence of the pneumococcus. Leroux sees in this arthritis after partial immunization a correspondence with the clinical experience that arthritis usually supervenes after a pneumonia has run its course or toward the end of an attack, when a condition of greater or less immunity has been acquired.

The symptoms of pneumococcic arthritis vary from pain and slight swelling limited to a single joint to an intense inflammatory œdema of the whole neighborhood of the articulation, or of a whole limb, with severe pain, heat, and redness, with abnormal mobility from destruction of the ligaments, and with grating of the bared surfaces of the bones. The fever is generally high (from 102° to 104°). The nature of the arthritis can be determined only by its association with pneumonia or other pneumococci infection, and by bacteriological examination of the joint contents. In cases that recover progress is slow, and the function of the joint is generally permanently impaired.—*Therapeutic Gazette.*

OINTMENT OF OXIDE ZINC, 10 PER CENT. ℞.—Benzoated lard, 8 ounces; spermaceti, ½ drachm; white wax, 1 drachm. Mix and add: Zinc oxide, 407 grains; starch, 60 grains. Stir until cold. The starch should be rubbed thoroughly and sifted before it is used. This formula will make a very smooth ointment, free from all grit, and of the same strength as the *United States Pharmacopœia.—Bull. Pharm.*

